# Implicit Attitudes toward the Self Over Time in Chinese Undergraduates

**DOI:** 10.3389/fpsyg.2017.01914

**Published:** 2017-10-31

**Authors:** Qing Yang, Yufang Zhao, Lili Guan, Xiting Huang

**Affiliations:** ^1^Key Laboratory of Cognition and Personality, Ministry of Education, School of Psychology, Southwest University, Chongqing, China; ^2^School of Psychology, Northeast Normal University, Changchun, China

**Keywords:** temporal self-appraisal, self, implicit attitudes, subjective temporal trajectory, implicit association test, Go/No-go association task

## Abstract

Although the explicit attitudes of Chinese people toward the self over time are known (i.e., past = present < future), little is known about their implicit attitudes. Two studies were conducted to measure the implicit subjective temporal trajectory (STT) of Chinese undergraduates. Study 1 used a Go/No-go association task to measure participants’ implicit attitudes toward their past, present, and future selves. The obtained implicit STT was different from the explicit pattern found in former research. It showed that the future self was viewed to be identical to the present self and participants implicitly evaluated their present self as better than the past self. Since this comparison of the past and present selves suggested a cultural difference, we aimed to replicate this finding in Study 2. Using an implicit association test, we again found that the present self was more easily associated with positive valence than the past self. Overall, both studies reveal an implicitly inclining-flat STT (i.e., past < present = future) for Chinese undergraduates. Implications of this difference in explicit-implicit measures and the cultural differences of temporal self appraisals are discussed.

## Introduction

The self exists on a continuum that originates in the past, lives in the present, and extends into the future (James, 1890/1950; Erikson, 1959). Attitudes about the past, present, and future can form a subjective temporal trajectory (STT), revealing how people look at and compare their self over the course of their life. Previous studies (e.g., Ryff, 1991; Wilson and Ross, 2001; Busseri, 2013; Liu and Huang, 2015) have mainly used explicit measures (e.g., questionnaires or self-descriptive judgments) to probe the STT in response to participants’ deliberate and reflective thoughts. Those studies found an inclining STT (i.e., past < present < future) in Western cultures (Busseri, 2013) and a flat-inclining STT (i.e., past = present < future) in Eastern cultures (Liu and Huang, 2015).

Until today however, few is known about how people implicitly and automatically think about their past, present, and futures. Given that a person’s implicit attitude is often independent of the explicit attitude toward the same attitude target (Fazio and Olson, 2003; Gawronski and Payne, 2011), the implicit STT might differ from the explicit STT. Learning about the implicit attitudes toward temporal selves can help to understand human motives, which are not easily captured via explicit measures. For instance, a comparison between implicit attitudes about the present and past selves can provide insights on how people might (implicitly) keep positive feelings (e.g., feelings of self-improvement or self-esteem) without (explicitly) violating the culturally shared social desirability (e.g., modesty). Thus, the present study measured Chinese participants’ implicit attitudes toward the self over time, and probed potential implicit–explicit differences.

### Explicit Attitudes toward the Self Over Time

In Western cultures, when people report their feelings on the past, present, and future, they rate themselves on average, better in the present than the past, and expect the future to be better than the present. This inclining STT (i.e., past < present < future) has been shown regarding people’s evaluations of their personality attributes (e.g., Wilson and Ross, 2001; Kanten and Teigen, 2008), physical attractiveness (e.g., Haddock, 2006), and psychological well-being (e.g., Ryff, 1991; Busseri, 2013). For instance, when college students evaluated their past, present, and future on several personality attributes (e.g., self-confident, pleasant), they rated positive attributes to describe themselves now more highly than positive attributes to describe themselves in the past (Wilson and Ross, 2001; Kanten and Teigen, 2008), and expected better in the future than in the present (Kanten and Teigen, 2008). Even for physical evaluation, people generally judged their present selves more physically attractive than their past selves, and expected their future attractiveness would be higher than their present attractiveness, especially for those who cared about their attractiveness (Haddock, 2006). Moreover, young and middle-aged adults reported they were experiencing a higher level of life satisfaction than in the past, and also expected the future to be happier than the present (Ryff, 1991; Staudinger et al., 2003; Busseri et al., 2009a,b, 2012; Busseri, 2013).

One straightforward explanation for the upward STT stems from life span theory, and indicates that people can perceive their improvement across different times in their lives in an accurate sense (Ryff, 1991; Fleeson and Heckhausen, 1997). That is, people can track their changes and experience their improvements because they truly exist. Another explanation is from the implicit theories of development (McFarland et al., 1992) that people hold culturally shared intuitive perspectives which “imply that life will get better and better” (Ross and Newby-Clark, 1998, p. 148), though sometimes people may not genuinely improve across time. A third explanation is based on the temporal self-appraisal theory (TSA; Wilson and Ross, 2001; Ross and Wilson, 2002) that people evaluate their past self in a way that serves the self-enhancement motive. People usually view their current selves more favorably than the past so that they can downwardly compare with it to maintain positive self-esteem. The self-enhancement motive can drive people to subjectively derogate the past self even in the absence of actual improvement of the present self (Wilson and Ross, 2001).

The upward STT drawn from previous studies have mainly used samples from Western cultures (e.g., Americans, Canadians). However, samples from Eastern cultures (e.g., Chinese, Japanese) have reported a somewhat different STT pattern.

The main difference across the two cultures occurs when comparing the present with the past. It seems that the past self is more valued and respected by people influenced by Eastern culture (Wang and Conway, 2004; Ross et al., 2005; Ji et al., 2009; Kim et al., 2012). When asking European and Asian Americans to think positively (vs. negatively) about their present, both groups reported higher life satisfaction (Kim et al., 2012). However, when thinking positively (vs. negatively) about their past, only Asian Americans reported higher life satisfaction. Canadian undergraduates described themselves more favorably at the present than in the past, but Japanese participants provided equally favorable or unfavorable descriptions about their present and past (Ross et al., 2005). Thus, it seems that people from Eastern cultures value their past and evaluate their present and past as equal (i.e., past = present).

The pattern is similar in the two cultures when comparing the present with the future (i.e., present < future). When simultaneously comparing the three temporal selves by asking Chinese college students to rate themselves on positive and negative adjectives, participants evaluated the past and present selves equally, but evaluated the future self more highly (Luo et al., 2010; Liu and Huang, 2015). Moreover, Chinese college students reported equal life satisfaction in their present and past, but expected higher life satisfaction in the future (Liu and Huang, 2015).

Overall, people from both Western and Eastern cultures have similar STTs when comparing the present with the future (i.e., present < future). However, they have a different pattern when comparing the past with the present (i.e., past < present in Western culture; but past = present in Eastern culture).

### Implicit Attitudes toward the Self Over Time

To summarize, we have reviewed the explicit attitudes of people toward their self over time and found a cultural difference in their STT patterns. However, few studies have investigated implicit STT patterns; consequently, little knowledge exists about how people intuitively and spontaneously judge their past, present, and future without using deliberate thoughts and reasoning. Studies often found different patterns for the same attitude target via explicit and implicit measures (Fazio and Olson, 2003; Gawronski and Payne, 2011). For example, self-esteem measured with the implicit association test (IAT) typically weakly correlated with that measured via self-reporting tools (Greenwald and Farnham, 2000; Jordan et al., 2003).

In the same vein, people’s implicit attitudes toward the self across time can be distinct from what they report in explicit measures. To our knowledge, there was one study that revealed such a difference. Peetz et al. (2014) asked undergraduates in Canada to report their explicit attitudes toward their present and future selves using the Rosenberg Self-Esteem Scale (Rosenberg, 1965), which measures explicit self-esteem. These participants also performed IATs to measure their implicit attitudes toward the present and future (i.e., implicit present/future self-esteem). They found that although participants expressed more positive explicit self-esteem toward the future than the present (i.e., explicit: present < future), their future implicit self-esteem was not enhanced compared with that of the present (i.e., implicit: present = future).

This explicit–implicit difference might be because explicit future self-esteem is vulnerable to social desirability and wishful thinking, potentially influencing Western people to explicitly report the future better than the present. Instead, implicit attitudes about the future are less influenced by social desirability, and are more affected by how often people automatically think about positive and negative aspects of the future (Peetz et al., 2014). Spontaneous thoughts about the future might be composed of both positive (e.g., graduation in 1 year) and negative (e.g., worry about getting a job after graduation in 1 year) valences. Thus, implicit attitudes about the future might not be as positive as those of the present when ratings from explicit measures are considered.

### The Present Research

However, since the study by Peetz et al. (2014) did not include attitudes about the past self, there is still no clear and integrated implicit STT. We aim to fill the research gap by investigating Chinese’ implicit attitudes toward the past, present and future selves. Given that the implicit attitudes are usually distinct with the explicit ones toward same attitude targets (e.g., Peetz et al., 2014), we predict that the pattern of implicit STT of Chinese participants might be different from the explicit one found in previous research (e.g., Liu and Huang, 2015). The integrated implicit STT, with its potential differences with the explicit STT, can provide a more comprehensive understanding of how people think about and evaluate their self over the course of life.

In Study 1 (using the Go/No-go association task; GNAT), we simultaneously compare the past, present, and future selves, to draw an implicit STT to compare with the explicit one found in previous research (i.e., past = present < future; Ross et al., 2005; Luo et al., 2010; Liu and Huang, 2015). We then discuss the implicit STT with consideration to the implicit–explicit difference and culture difference. Based on this discussion, Study 2 focused on replicating the implicit pattern between the present and past selves, using an IAT.

## Study 1

### Methods

#### Ethics Statement

The present research was approved by the Human Research Ethics Committee of Southwest University, and all participants of both studies provided written informed consent in accordance with the Declaration of Helsinki. The protocol was approved by the Human Research Ethics Committee of the Southwest University.

#### Participants

Seventy-seven Chinese undergraduate students (46 females and 31 males, mean age = 20.7 years, age range = 17–23 years) from Southwest University participated in this study.

#### Procedure

This GNAT task was introduced as a categorization task in which participants had to categorize different items (words) according to instructions. Five kinds of stimuli were used: past words, present words, future words, positive words, and negative words (all in Chinese). The past, present, and future words included only one word in each category: “past me” (“
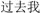
”), “present me” (“
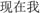
”), or “future me” (“
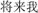
”), respectively. The positive and negative items consisted of ten words each (e.g., positive words: confident, smart, optimistic; negative words: unsuccessful, pessimistic, conflicted) that were selected from an established pool of Chinese personality-trait adjectives (Huang and Zhang, 1992). These words were all commonly used and had the same word length in Chinese. Moreover, the positive and negative words differed in valence, *t*(27) = 31.50, *p* < 0.001, *d* = 1.94, but not in arousal, *t*(27) = -1.79, *p* = 0.085, *d* = 0.44, according to a pretest with another 28 college participants (10 females).

After practice, participants completed six blocks (in random order) during the formal task. In each block, participants were required to identify if the stimuli (e.g., “smart”) belonged to a target association (e.g., past words + positive words). For example, in one block, participants had to identify if “smart” belonged to any of the category of past words or positive words (the answer is yes in this example). There were six associations (six blocks) in total: past + positive, past + negative, present + positive, present + negative, future + positive, and future + negative.

**FIGURE 1:**
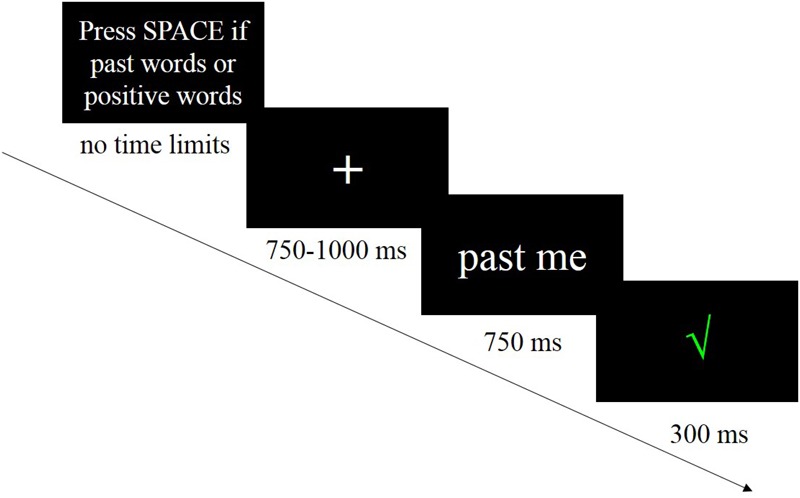
A trial in one of the six blocks. After understanding the instruction in this block, participants started the trials. A total of 40 trials in each block.

For each trial (see **Figure 1** for the procedure), a fixation point was presented for 750–1000 ms at the center of the screen, then a stimulus was presented. If a stimulus belonged to the target association (the stimulus was called a “signal”), then the participant pressed the SPACE key as quickly and accurately as possible within 750 ms (go response). If not (the stimulus was called “noise”), the participant did not press any button (no-go response). It was then followed by a feedback for 300 ms. We set 750 ms as the response deadline because Nosek and Banaji (2001) suggested a 500–850 ms range to minimize possible ceiling or floor effects in response accuracy. A pretest also showed 750 ms was appropriate for most participants. Participants completed 40 trials in each block, which included 20 signals (go responses) and 20 noises (no-go responses). For example, in the past + positive block, this would randomly show 10 past words (i.e., “past me” 10 times) and 10 different positive words as signals, while it would show five present words (i.e., “present me” for five times), five future words (i.e., “future me” for five times), and 10 different negative words as the noises.

### Results and Discussion

Following the standard GNAT procedure (Nosek and Banaji, 2001), sensitivity (d’), which indicates the ability to discriminate signals from noises, was calculated. Greater d’ represents that the target association (e.g., present words + positive words) was tighter. For example, greater d’ in this association represented that individuals had a more positive attitude toward the present self. First, in each block, we calculated the proportion of hits (correct “go response” for signals) and false alarms (incorrect “go response” for noises) and converted each to *z*-scores. Second, we calculated the difference (*z*-score values) between the hits and false alarms as d’ (see **Table 1**).

**Table 1:** Means and standard deviations (in parentheses) of d’ by temporal selves and attribute valence.

	Past self	Present self	Future self
Positive	2.83 (0.89)	3.49 (0.81)	3.56 (0.79)
Negative	3.80 (0.66)	3.33 (0.78)	3.41 (0.78)

**FIGURE 2:**
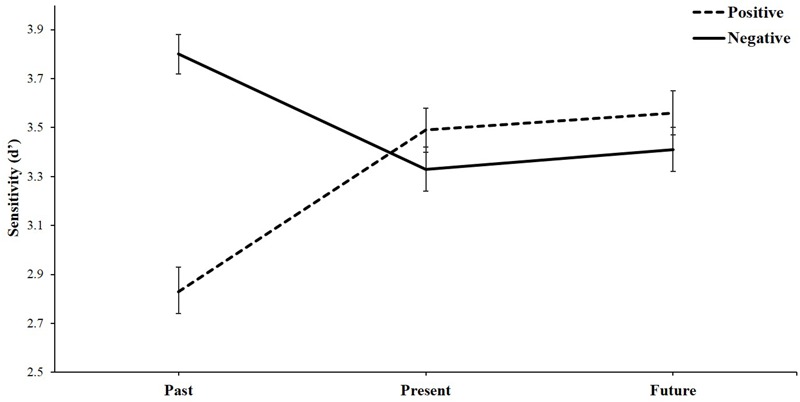
Sensitivity d’ as a function of attribute valence and temporal selves. Error bars of all figures indicate standard errors (SEs).

A 3 (temporal selves: past vs. present vs. future) × 2 (attribute valence: positive words vs. negative words) repeated measures analysis of variance (ANOVA) was conducted, with d’ as the dependent measure, as shown in **Figure 2**. The main effect of attribute valence was significant, indicating that negative words (*M* = 3.51, *SE* = 0.07) were more easily identified than positive words (*M* = 3.29, *SE* = 0.07), *F*(1,76) = 11.74, *p* = 0.001, η^2^ = 0.134. Moreover, the interaction between temporal selves and attribute valence was significant, *F*(2,75) = 34.02, *p* < 0.001, η^2^ = 0.476. We probed this interaction with simple effects analyses.

For positive words, we observed a significant effect, *F*(2,75) = 24.10, *p* < 0.001, η^2^ = 0.391. The present self (*M* = 3.49, *SE* = 0.09) was more closely associated with these words than the past self (*M* = 2.83, *SE* = 0.10), *t*(76) = 5.82, *p* < 0.001, *d* = 1.33. The future self (*M* = 3.56, *SE* = 0.09) was also more closely associated with these words than the past self (*M* = 2.83, *SE* = 0.10), *t*(76) = 6.65, *p* < 0.001, *d* = 1.52. The present and future selves were not significantly different, *t*(76) = -0.69, *p =* 0.493, *d* = -0.16.

However, for negative words, there was a reverse trend, *F*(2,75) = 14.22, *p* < 0.001, η^2^ = 0.275. The past self (*M* = 3.80, *SE* = 0.08) was more closely associated with these words than the present self (*M* = 3.33, *SE* = 0.09), *t*(76) = 5.11, *p* < 0.001, *d* = 1.16, and the future self (*M* = 3.41, *SE* = 0.09), *t*(76) = 4.08, *p* < 0.001, *d* = 0.93. The present and future selves were not significantly different, *t*(76) = -0.78, *p =* 0.437, *d* = -0.18.

Negative words (*M* = 3.80, *SE* = 0.08) were more closely associated with the past self than positive words (*M* = 2.83, *SE* = 0.10), *F*(1,76) = 74.84, *p* < 0.001, η^2^ = 0.496, suggesting that the past self was perceived as generally negative by college students. Negative and positive words were not significantly different in their associations with the present self, *F*(1,76) = 2.27, *p* = 0.136, η^2^ = 0.029. Nor were negative and positive words significantly different in their associations with the future self, *F*(1,76) = 2.58, *p* = 0.112, η^2^ = 0.033. These findings suggest that implicit attitudes toward the present and future might be associated with equally positive and negative valences. No other significant effects were found.

**FIGURE 3:**
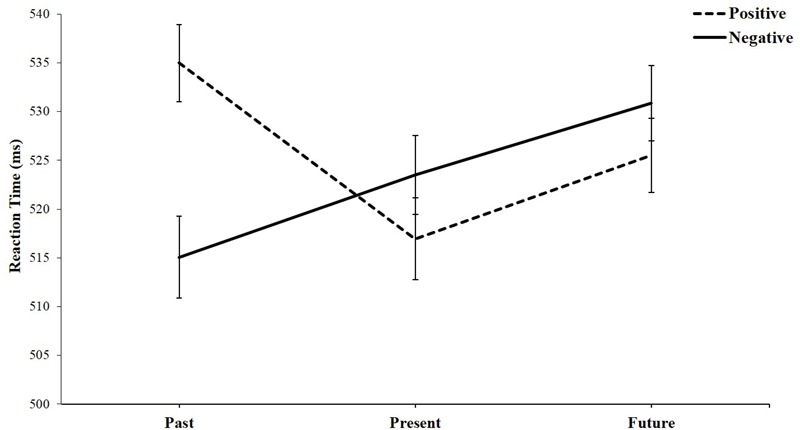
Reaction times (ms) of hit responses as a function of attribute valence and temporal selves.

Additionally, we computed reaction times (RTs) of “go responses” (i.e., hits responses) in six blocks. A same repeated measures ANOVA was performed. Similarly, a significant interaction effect was found: *F*(2,75) = 12.56, *p* < 0.001, η^2^ = 0.251. As shown in **Figure 3**, simple effects trends were generally consistent with the d’ measure.

Specifically, for positive words, we found a significant effect, *F*(2,75) = 8.72, *p* < 0.001, η^2^ = 0.189. Participants responded faster when their present self (*M* = 516.95 ms, *SE* = 4.20) was associated with these words than their past self (*M* = 534.99 ms, *SE* = 3.94), *t*(76) = 4.20, *p* < 0.001, *d* = 0.96. Participants also responded faster when their future self (*M* = 525.53 ms, *SE* = 3.80) was associated with these words than their past self, *t*(76) = 2.51, *p* = 0.014, *d* = 0.57. Moreover, participants responded slightly faster when their present self was associated with these words than their future self, *t*(76) = 2.40, *p* = 0.019, *d* = 0.55.

For negative words, this trend was reversed, *F*(2,75) = 8.97, *p* < 0.001, η^2^ = 0.193. Participants responded faster when their past self (*M* = 515.07 ms, *SE* = 4.21) was associated with these words than their present self (*M* = 523.49 ms, *SE* = 4.05), *t*(76) = 2.10, *p* = 0.039, *d* = 0.48, and than their future self (*M* = 530.86 ms, *SE* = 3.85), *t*(76) = 4.27, *p* < 0.001, *d* = 0.97. Participants responded marginally faster when their present self was associated with these words than their future self, *t*(76) = 1.98, *p* = 0.051, *d* = 0.45.

Taking a different perspective, negative words (*M* = 515.07 ms, *SE* = 4.21) led to faster responses than positive words (*M* = 534.99 ms, *SE* = 3.94) when they were associated with the past self, *F*(1,76) = 22.35, *p* < 0.001, η^2^ = 0.227; however, this was not the case when they were associated with the present self, *F*(1,76) = 2.88, *p* = 0.094, η^2^ = 0.036, or the future self, *F*(1,76) = 1.93, *p* = 0.168, η^2^ = 0.025.

Overall, these results consistently suggest an implicit “past < present = future” STT in Chinese undergraduates: the present and future selves are implicitly perceived as more positive than the past self, but the present and future selves are perceived as equally positive. These results also suggest that Chinese undergraduates have an unequivocally negative attitude about their past (i.e., negative > positive), but more balanced (or neutral) feelings about their present and future (i.e., positive = negative).

In sum, considering the STT patterns across different cultures and measures, both similarities and differences exist. **Table 2** summarizes the STT patterns that have been found in the previous and present research. The two cultures show a similar pattern when comparing the present and future selves regardless of measures. With explicit measures, both cultures expect the future to be better than the present (i.e., future > present). But with implicit measures, the future self is not perceived as better than the present self in both cultures (i.e., future = present).

**Table 2:** Subjective temporal trajectory (STT) patterns in Western and Eastern cultures using explicit and implicit measures.

	Western culture	Eastern culture
Explicit measures	Past < Present < Future	Past = Present < Future
Implicit measures	Present = Future^∗^	Past < Present = Future

*^∗^To our knowledge, there has no research comparing the present and past selves in western culture context with implicit measures.*

The main difference between Eastern and Western cultures manifests when comparing the present with the past. With explicit measures, Western people perceive their present as better than the past (i.e., present > past), whereas Eastern people view their present and past selves equally (i.e., present = past). With implicit measures, research conducted in Western culture is still lacking, and our research reveals that Eastern people implicitly perceive their present as better than their past (i.e., present > past).

In Study 2 we use an IAT to try to replicate the “present > past” finding. Because the pattern of comparison between the present and future has been quite consistent across cultures even when using different measures, we do not compare them in Study 2.

## Study 2

The typical IAT paradigm (Greenwald et al., 1998, 2003) measures the automatic associations between target concepts (e.g., present me and past me) and attributes (e.g., positive items and negative items). If the speed of response to one combination (e.g., present me + positive items and past me + negative items) is faster than the other (e.g., past me + positive items and present me + negative items), this indicates that the former combination (called a compatible combination) was stronger than the latter (called an incompatible combination). In our case, this would imply that the implicit attitude of the “present me + positive items” is more consistent than the association of “past me + positive items,” or the “past me + negative items” association is more consistent than the association “present me + negative items.” That is, the present self is perceived as more positive (and less negative) than the past self.

### Methods

#### Participants

Forty-one undergraduate students (native Chinese speakers) from the Southwest University (37 females and 4 males, mean age = 21.1 years, age range = 18–23 years) participated in this study.

#### Procedure

This task was introduced as a categorization task in which participants had to categorize different items according to instructions. Like the typical IAT paradigm (Greenwald et al., 1998, 2003), four kinds of stimuli were used: present me items, past me items, positive items, and negative items. In contrast to Study 1, in Study 2, the “present me” and “past me” items consisted of 10 words each, which were commonly used but had different descriptions in Chinese (e.g., present me: 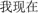, 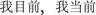; past me: 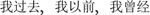). The positive and negative words consisted of 10 words each that were used in Study 1.

There were seven blocks of trials, which consisted of five practice blocks and two critical blocks (see **Table 3** for the design). Blocks 3/4 and Blocks 6/7 were used for data collection (practice trials in Blocks 3 and 6 were used to increase power, as suggested by Greenwald et al., 2003). Before each block, the participants were informed which kind of items they had to categorize and which keys (F or J) they should press. The labels of the required items remained on the top of the screen during each block. For each trial, a fixation was presented for 750 – 1000 ms at the center of the screen. Then a stimulus was presented until the participant pressed a key (F or J) on a standard keyboard, which was followed by feedback for 300 ms (No feedback was given in the critical blocks. Instead, the screen was blank for 300 ms.). Participants were asked to respond quickly and accurately. Our main interest focused on the difference in RTs between the compatible combinations of Blocks 3/4 and the incompatible combinations of Blocks 6/7. The order of the compatible and incompatible combination blocks and the keys assigned to different items were counterbalanced across participants.

**Table 3:** The design of the implicit association test.

Block	Labels1 (key F)	Labels2 (key J)
(1) (practice block, 20 trials)	Past me items	Present me items
(2) (practice block, 20 trials)	Negative items	Positive items
(3) (practice block, 40 trials)	Past me + Negative items	Present me + Positive items
(4) (critical block, 40 trials)	Past me + Negative items	Present me + Positive items
(5) (practice block, 20 trials)	Positive items	Negative items
(6) (practice block, 40 trials)	Past me + Positive items	Present me + Negative items
(7) (critical block, 40 trials)	Past me + Positive items	Present me + Negative items

### Results and Discussion

#### Preliminary Analyses

Response accuracies (ACCs) and RTs of four associations (past + negative, present + positive, past + positive, and present + negative) were computed and their means are listed in **Table 4**. The means of ACCs and RTs in all four associations were used for a one-way ANOVA.

**Table 4:** Means and standard deviations (in parentheses) of response accuracies and reaction times (ms) for four associations in the IAT.

	Past + Positive	Past + Negative	Present + Positive	Present + Negative
Response Accuracy	0.913 (0.07)	0.960 (0.04)	0.968 (0.03)	0.910 (0.08)
Reaction Time (ms)	954.45 (219.55)	701.49 (138.97)	689.27 (133.12)	966.24 (183.96)

**FIGURE 4:**
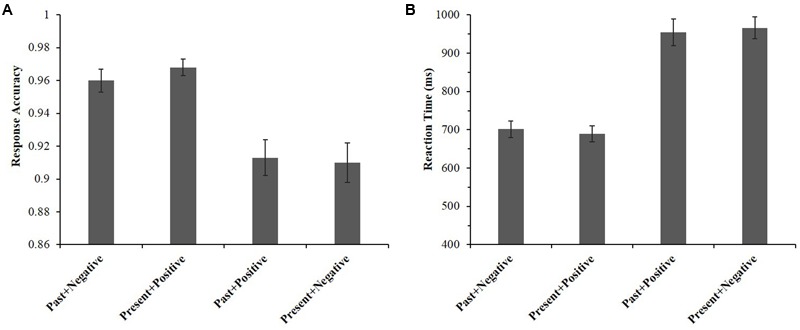
Comparisons of response accuracies **(A)** and reaction times **(B)** among four associations during the IAT.

For ACC, a significant effect was found, *F*(3,38) = 16.05, *p* < 0.001, η^2^ = 0.559; see **Figure 4A**. Specifically, participants had significantly higher ACC in the past + negative association (*M* = 0.960, *SE* = 0.007) compared to the past + positive association (*M* = 0.913, *SE* = 0.011), *t*(40) = 4.70, *p* < 0.001, *d* = 1.47, and the present + negative association (*M* = 0.910, *SE* = 0.012), *t*(40) = 5.56, *p* < 0.001, *d* = 1.74. Similarly, participants also showed significantly higher ACC in the present + positive association (*M* = 0.968, *SE* = 0.005) than in the past + positive association, *t*(40) = 5.50, *p* < 0.001, *d* = 1.72 and the present + negative association, *t*(40) = 5.90, *p* < 0.001, *d* = 1.84. Neither the difference between past + negative and present + positive associations, nor the difference between past + positive and present + negative associations reached a significant level, *ts*(40) < 1.30, *p* > 0.990, *d* < 0.41. These results suggest that the task was simpler for participants in compatible associations (e.g., present + positive) than in incompatible associations (e.g., present + negative).

For RT, a significant effect was also found, *F*(3,38) = 54.09, *p* < 0.001, η^2^ = 0.810; see **Figure 4B**. Similar to the trends observed in ACC, participants responded significantly faster in the past + negative association (*M* = 701.49 ms, *SE* = 21.70) than in the past + positive association (*M* = 954.45 ms, *SE* = 34.29), *t*(40) = 10.96, *p* < 0.001, *d* = 3.42 and the present + negative association (*M* = 966.24 ms, *SE* = 28.73), *t*(40) = 12.57, *p* < 0.001, *d* = 3.93. Furthermore, the RT in the present + positive association (*M* = 689.27 ms, *SE* = 20.79) was significantly faster than in the past + positive association, *t*(40) = 9.81, *p* < 0.001, *d* = 3.06 and than in the present + negative association, *t*(40) = 12.55, *p* < 0.001, *d* = 3.92. Neither the differences between past + negative and present + positive associations, nor the differences between past + positive and present + negative associations were significant, *ts*(40) < 1.35, *p* > 0.990, *d* < 0.43. This revealed that participants responded faster in compatible associations (e.g., present + positive) than in incompatible associations (e.g., present + negative).

#### Main Analyses

More importantly, we took a general perspective and investigated the RTs difference between compatible combinations (past + negative combined with present + positive) and incompatible combinations (past + positive combined with present + negative). Following Greenwald et al. (1998, 2003), trials with correct responses in Blocks 3/4 and Blocks 6/7 were analyzed, with the first two trials of each block discarded. Values below 300 ms were recoded as 300 ms and values above 3000 ms were recoded as 3000 ms. The mean RTs in compatible combination trials (past + negative and present + positive) and incompatible combination trials (past + positive and present + negative) were calculated.

**FIGURE 5:**
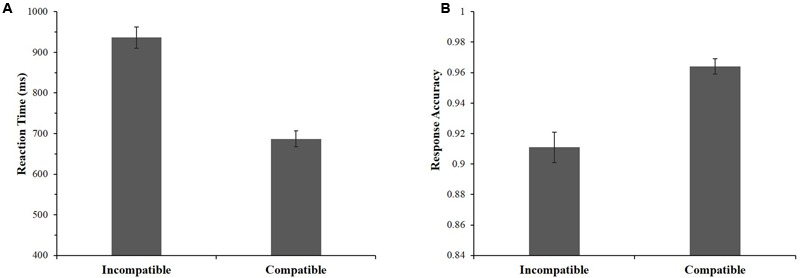
Reaction time **(A)** and response accuracy **(B)** differences between compatible combinations (past + negative combined with present + positive) and incompatible combinations (past + positive combined with present + negative).

We conducted a paired-sample *t*-test to examine the difference between compatible and incompatible combinations. We found that RTs were significantly faster for compatible combinations (*M* = 686.93 ms, *SD* = 125.60) compared to incompatible combinations (*M* = 936.29 ms, *SD* = 169.37), *t*(40) = 14.57, *p* < 0.001, *d* = 4.55, as shown in **Figure 5A**. This result confirmed that the present self was implicitly regarded as more positive (i.e., less negative) than the past self (i.e., present > past).

Additionally, we computed the ACCs for both combinations. Participants had a significant higher accuracy for compatible combinations (*M* = 0.964, *SD* = 0.03) than for incompatible combinations (*M* = 0.911, *SD* = 0.06), *t*(40) = 7.14, *p* < 0.001, *d* = 2.23, as shown in **Figure 5B**. This suggested that compatible combinations were generally simpler for participants than incompatible combinations.

Overall, these findings are consistent with the findings of Study 1, in which the present self is implicitly perceived as better than the past self. The implications are discussed in the next section.

## General Discussion

Using implicit measures, two studies examined Chinese undergraduates’ implicit attitudes toward the self over time. Chinese undergraduates implicitly have a more positive attitude toward their present and future, compared with their attitude about the past. But they implicitly perceive the present and future as equally positive. Overall, there is a “past < present = future” implicit STT in Chinese undergraduates. Additionally, Study 1 also revealed that Chinese undergraduates generally have a negative attitude toward their past (i.e., negative > positive), but they have more balanced (neutral) feelings about both their present and future (i.e., negative = positive).

### Comparison of Attitudes about the Present and Past Selves

The present study found that with implicit measures, Chinese college students indicated they are much better in the present than they were in the past. Such an implicit pattern can likely be explained by depreciating the past, by enhancing of the present, or by both of these processes. Previous research that used explicit measures (Luo et al., 2010; Liu and Huang, 2015) found that Chinese college participants endorsed positive over negative attributes at an overwhelming rate, even when evaluating their past (i.e., positive > negative), our research (Study 1) showed that they had an unequivocally negative attitude toward their past (i.e., negative > positive), and a balanced attitude toward the present (i.e., negative = positive). It therefore seems more likely that at least to some extent, participants implicitly depreciated their past self.

If Chinese participants used self-depreciating, it is an interesting question what motivation might underlie this process. One possibility is that the evaluation of the past is based on implicit theories of development (McFarland et al., 1992). Even in Eastern cultures, people share a general idea that individuals will and should improve with age at a number of traits. Young adult participants might use theories of development to infer their past standing on some specific attribute (e.g., depreciating the past to feel like having improved in the present), even if it remains unclear whether they truly have improved or not. A further possibility has been addressed from TSA (Wilson and Ross, 2001). TSA suggests that people usually derogate their past and thus can downwardly compare with their past to maintain a positive self-regard for their present self. This can also happen even if actual improvement is non-existent. Future research is recommended to clarify the underlying motivation of implicit appraisals of past and present selves.

Moreover, although Chinese participants implicitly feel better than their remembered past, they seem unwilling to explicitly show this. This might be because explicit self-reports usually follow a socially more acceptable manner (Kobayashi and Greenwald, 2003). Eastern cultures emphasize modesty (e.g., downplaying accomplishments) over self-presentation (Bond et al., 1982; Markus and Kitayama, 1991; Kurman, 2003). But even if Chinese participants implicitly believe that they have improved since the past, they may not feel comfortable to explicitly or directly claim this, as this would conflict with social desirability, which discourages self-presentation. However, modesty impacts less on how people implicitly express their feelings (Cai et al., 2007). For example, Shi et al. (2017) found that modest priming could decrease Chinese participants’ self-positivity bias when using relatively explicit measures (i.e., self-descriptive judgments about trait words), while this did not influence the self-positivity bias measured in a relatively implicit way (i.e., response times for the judgment of trait words). Thus, social desirability (e.g., modesty) might be one of the important factors, which can determine the implicit-explicit difference of appraisals of the past and present selves. In addition, it remains unclear whether such an implicit-explicit discrepancy also exists in Western cultures, given that the implicit evidence is still missing. Future research should therefore explore this issue, which should provide a comprehensive picture of implicit-explicit discrepancy of appraisals of the past and present selves across different cultures.

### Comparison of Attitudes about the Present and Future Selves

Although a cultural difference is implied when comparing present with the past selves, a consistent trend in both cultures arises when the present selves are compared to future selves. When using deliberative and reflective thoughts (i.e., self-reports), people in both cultures expect their future to be better than the present. But when using automatic and intuitive thoughts (i.e., implicit measures) they deem their present and future generally equal. It is not surprising that people in both cultures explicitly report their future to be better than the present, because thoughts about the future are less restricted by reality than the present (Robinson and Ryff, 1999; Newby-Clark and Ross, 2003), and dreaming about a bright future is encouraged by both cultures. Consequently, people more easily engage in wishful thinking and are usually very optimistic about their future (Robinson and Ryff, 1999; Newby-Clark and Ross, 2003; Liu and Huang, 2015). They tend to report the present and future in a manner reflecting the idea that “life will get better and better” (Ross and Newby-Clark, 1998, p. 148).

However, implicit attitudes about the future are conceptualized as cognitive associations of the future self with positive or negative valence (Peetz et al., 2014). The implicit future attitude is thus dependent on how often people automatically and spontaneously think about the positive and negative sides associated with their future self (Peetz et al., 2014; see also Ratliff and Nosek, 2010). Thinking about the future is often goal-directed (Karniol and Ross, 1996; Oettingen and Mayer, 2002). The successful goal-pursuit entails both expecting a bright future and thinking about the potential difficulties, whereas the goal-pursuit cannot be promoted and can even be hampered by relying on merely wishful thinking, such as fantasy (Oettingen and Mayer, 2002).

Thus, while people usually fantasize about a future more positive than the present in their deliberative thoughts, their implicit and intuitive attitudes about the future might often be composed of positive and negative valences. Our GNAT results confirmed this, suggesting that Chinese undergraduates have a balanced/neutral feeling about their future (and their present). When they associate bright things with their future (or present) life, they equally associate gloomy things with it. We believe this makes their implicit attitudes toward the present and future selves more balanced than their explicit attitudes. The future then is not implicitly believed to be much better than the present.

### Limitations and Future Directions

The present research is not without limitations. Future research might have some more interesting findings by addressing these issues. First, in terms of statistical power, although the effect sizes and statistical powers (1-β > 0.95) seemed satisfying in the main results, we acknowledge that the sample sizes can be larger and better with considering increasing the diversity of samples in future designs. Given that our participants are mainly young adults in college, and that STT is subject to different age stages (Ryff, 1991; Busseri, 2013) and can be influenced by dispositional traits (e.g., dispositional optimism; Busseri, 2013; Busseri et al., 2013), it is necessary to include different samples and consider individual differences in future studies to increase the generalizability of the present findings. Second, future research might want to investigate participants of different cultures, and use both explicit and implicit measures in the same experimental set, thus yielding a more comprehensive understanding of STTs across cultures.

## Conclusion

Using implicit measures, our research reveals that Chinese undergraduates evaluate their present and future to be about equal, and evaluate their present and future to be more positive than their past. We find an implicit “past < present = future” STT, which differs from the explicit “past = present < future” STT delineated in previous research (Luo et al., 2010; Liu and Huang, 2015). Our results also reveal some similarities and differences with attitudes toward the self over time in people from Western cultures. These findings help to both understand and explain cultural differences as well as universalities in human motives and in their behaviors.

## Author Contributions

QY and XH conceived the research, QY conducted the experiments and analyzed the data, QY, YZ, and LG wrote and revised the manuscript. XH supervised the project. All authors reviewed the manuscript.

## Conflict of Interest Statement

The authors declare that the research was conducted in the absence of any commercial or financial relationships that could be construed as a potential conflict of interest.
